# Anti-ALDOA antibody: a novel diagnostic-associated autoantibody in myasthenia gravis

**DOI:** 10.3389/fneur.2025.1696583

**Published:** 2025-10-23

**Authors:** Mengdi Zhang, Yingna Zhang, Peipei Liu, Qingye Gao, Qian Liu, Xinyue Zhou, Ruichen Liu, Xue Zhao, Jing Zhang, Jia Hu, Haiyan Dong, Jing Liu, Junhong Yang, Zhe Ruan, Ting Chang, Jie Lv, Feng Gao

**Affiliations:** ^1^Department of Neuroimmunology, Henan Institute of Medical and Pharmaceutical Sciences, Zhengzhou University, Zhengzhou, China; ^2^Department of Encephalopathy, First Affiliated Hospital of Henan University of Chinese Medicine, Zhengzhou, China; ^3^Department of Neurology, Tangdu Hospital, The Fourth Military Medical University, Xi’an, China

**Keywords:** seronegative myasthenia gravis, clinical diagnosis, autoantibody, tissue-based assay, proteomics

## Abstract

This study identified novel autoantibodies in patients with seronegative myasthenia gravis (SNMG).Autoantibodies in SNMG sera were screened using a targeted binding assay (TBA). The target antigen was identified via immunoprecipitation and mass spectrometry, and validated by western blotting using a commercial antigen. A cell-based assay (CBA) employing HEK293 cells expressing the identified antigen was established to detect specific antibodies in 676 MG patients and 20 controls.Aldolase A (ALDOA) was identified as a novel autoantigen. Anti-ALDOA antibodies (ALDOA-Ab) demonstrated high diagnostic specificity in SNMG and were also detected in seropositive MG patients, with positivity rates of 4.89% in AChR-Ab’ and 2.86% in MuSK-Ab’ subgroups. ALDOA-Ab was predominantly of the IgG1 subclass. ALDOA-Ab may serve as a potential diagnostic biomarker for MG. Further studies are needed to investigate its pathogenic role.

## 1 Introduction

Myasthenia gravis (MG) is an antibody (Ab)-mediated autoimmune disease characterized by fluctuating muscle weakness and fatigability (1, 2). Anti-acetylcholine receptor (AChR) (3) antibody, anti-muscle-specific receptor tyrosine kinase (MuSK) (4) antibody, and anti-low-density lipoprotein receptor-related protein 4 (LRP4) (5) antibody have been shown to be associated with MG, with reported positivity rates of approximately 85% for AChR, 1–10% for MuSK, and 1–5% for LRP4. However, LRP4 antibodies are more common in AChR/MuSK-negative patients (6). Furthermore, other muscle-specific autoantibodies, such as those targeting the ryanodine receptor (RyR), titin, striational proteins, agrin, collagen XIII, and cortactin (7), have also been identified in subsets of MG patients, particularly those with thymoma or more severe disease phenotypes. While the precise pathogenic roles of these antibodies are not fully identified, they are often associated with specific clinical characteristics and are considered markers of disease severity or particular subtypes. The three autoantibodies are undetectable in the serum of approximately 15% of patients with MG, which is referred to as seronegative myasthenia gravis (SNMG) (8). The clinical diagnosis of SNMG depends on typical clinical features, responsiveness to cholinesterase inhibitors, and electrodiagnostic studies (repetitive nerve stimulation and single-fiber electromyography), which is easy to cause delayed diagnosis or misdiagnosis (9). In addition, serological typing using autoantibodies, namely AChR, MuSK, and LRP4, facilitates personalized treatment or drug selectivity in MG patients. The presence or absence of pathogenic autoantibodies in SNMG patients is still unclear. Hence, precise treatment of these patients remains unachievable with the currently available targeted therapies, which highlights the urgent need to explore whether the sera of SNMG patients contain the potential autoantibodies (6, 10–14).

In 2020, Hoffmann S et al. found complement deposits at the motor endplates of biopsied muscle tissues from SNMG patients (8). In our previous unpublished study, SNMG patients’ sera were co-incubated with rat gastrocnemius muscle tissues, which exhibited characteristic fluorescent signals under immunofluorescence staining, suggesting that autoantibodies targeting gastrocnemius muscle proteins may exist in the patients’ serum. In conclusion, SNMG patients are likely to have autoantibodies related to the disease, which have not yet been identified. Therefore, in this study, we used immunoprecipitation and mass spectrometry to screen for antibodies against fructose-bisphosphate ALDOA in the serum of SNMG patients and conducted a preliminary exploration of the relationship between the presence of the antibody and the clinical symptoms.

## 2 Samples and methods

### 2.1 Study population

The diagnostic criteria for myasthenia gravis is as follows: (1) clinical manifestations including the typical symptom of muscle weakness, which aggravated post-activity and improved after rest, (2), a positive neostigmine test, (3) compound muscle action potential (CMAP) amplitude reduction ≥ 10% in a low-frequency repetitive nerve stimulation (RNS) test, (4) a positive test for antibodies such as AChR-Ab, MuSK-Ab, or LRP4-Ab. The diagnosis of myasthenia gravis is confirmed if any one of the criteria (2–4, or) is met along with criterion 1. If the antibody is positive, then the patient is considered seropositive for MG.

Inclusion criteria for SNMG comprised the following: (1) typical clinical features; (2) positive neostigmine and/or RNS test findings; (3) repeated negative results for AChR (ELISA), MuSK, and LRP4 (CBA) antibodies; (4) no history of immunosuppressive therapy for ≥1 year; and (5) no history of thymectomy.

MG patients registered at Henan Province Neuroimmune Precision Diagnosis and Treatment Engineering Technology Research Center (HNETC) from January 2015 to March 2021 were retrospectively analyzed. A total of 676 MG patients were included in the study, comprising 20 SNMG patients and 656 SPMG patients. Among these, 613 were AChR-Ab^+^, 35 were MuSK-Ab^+^, and 8 were LRP4-Ab^+^.

A total of 20 patients with idiopathic inflammatory myopathies (IIM) served as disease controls. These patients were diagnosed based on the presence one or more of the following antibodies: Mi-2, Ku, PM-Scl100, PM-Scl75, Jo-1, SRP, PL-7, PL-12, EJ, or Ro-52 (detected using the EUROIMMUN Myositis Profile IgG blot kit, Lot D190606AD).

The study was approved by the ethics committee, and all enrolled patients consented to the collection of peripheral blood serum for the test and provided their clinical information by signing an informed consent form.

### 2.2 TBA assay and immunoprecipitation

Frozen sections of rat gastrocnemius muscle were incubated with serum from screening stage patients [diluted 1:10 in a solution of 0.5% triton X 100 in phosphate-buffered saline (PBS) buffer] for 2 h at 37 °C. The samples were then washed three times with PBS for 5 min each, with the addition of Alexa Fluor-488 goat anti-human Immunoglobulin G (IgG; 1:1,000 dilution in 0.5% triton X 100 in PBS buffer), incubated for 1 h at 37 °C and washed three times with PBS; and visualized under a fluorescence microscope (Nikon TS100-F inverted fluorescence microscope; Nikon Corporation, Tokyo, Japan). The patient sera demonstrated characteristic fluorescent signals on frozen tissue sections and were screened for immunoprecipitation. The immunoprecipitated product proteins were obtained for subsequent comparative validation.

### 2.3 SDS-PAGE separation and mass spectrometry identification

In total, 20 microliters (μL) of the immunoprecipitated product were electrophoresed on 10% sodium dodecyl sulfate polyacrylamide gel electrophoresis (SDS-PAGE) gel and stained with Coomassie brilliant blue G-250. Differential protein bands between the disease group and the healthy control (HC) group were selected and cut, following which, then the protein strips were sent to Jingjie Biotechnology Co., Ltd. (Hangzhou, China) for mass spectrometry identification.

### 2.4 Western blot validation

Commercialized target antigen (cloud clone) was purchased for immunoblotting validation. A total of 20Twenty microliters of the protein was electrophoresed on 10% SDS-PAGE gel, transferred to a polyvinylidene difluoride (PVDF) membrane, blocked for 1 h in 5% non-fat milk, and incubated with patient’s serum (1:100) overnight at 4 °C. Diluted (1:5,000) HRP-conjugated anti-human antibody was used for the detection of primary antibodies for 60 min, followed by the use of an enhanced chemiluminescence (ECL) kit for further analysis.

### 2.5 HEK 293 cells expressing target antigen

HEK 293 cells were cultured in high-glucose Dulbecco’s Modified Eagle Medium (DMEM) containing 10% fetal bovine serum and inoculated in 48-well cell culture plates. The cells were then transfected with either the pcDNA3.1-ALDOA-EGFP plasmid or the pcDNA3.1-EGFP-C control plasmid (YouBio, NM000034) using Lipofectamine 2000 reagent according to the manufacturer’s instructions. Forty-eight hours post-transfection, the cells were fixed with 4% paraformaldehyde for 20 min and washed three times with PBS.

### 2.6 CBA assay

The cell-based assay (CBA) involved incubation of serum (1/10 dilution in 1% bovine serum albumin in 0.5% Triton X-100) with transfected cells. After 1.5 h, the cells were washed three times with 0.25% Triton X-100, followed by the addition of anti-human IgG (fluorescent secondary antibody) labeled with Alexa Fluor-568 (1/1,000 dilution in 0.5% Triton X-100). The cells were observed using TS100 microscopy (Nikon TS100-F inverted fluorescence microscope; Nikon Corporation, Tokyo, Japan) by two or three independent observers. The Alexa Fluor-568-labeled goat anti-human IgG antibody resulted in red fluorescence, and the ALDOA-EGFP fusion protein produced green fluorescence. When ALDOA-Ab was present in the serum, the red fluorescence and green fluorescence overlapped to exhibit yellow fluorescence.

Identification of antibody subtypes: 48-well cell plates transfected with pcDNA3.1-ALDOA-EGFP plasmid were fixed and blocked. In total, 100 μL of serum from ALDOA-Ab single-positive MG patients diluted 1:10 with 0.5% Triton X-100 was added per well and incubated for 1.5 h at 37 °C and then washed three times. A total of 100 μL of mouse anti-human IgG subtype antibody (1:250 dilution) was added and incubated for 1.5 h at room temperature and then washed. Alexa Fluor-568 goat anti-mouse IgG antibody (1:1,000 dilution) was added and incubated for 1.5 h at 37 °C, and then washed three times. Finally, the cells were observed using TS100 microscope (Nikon, Japan).

## 3 Results

### 3.1 Clues to the presence of potential autoantibodies in the serum of SNMG patients

**Figure 1:**
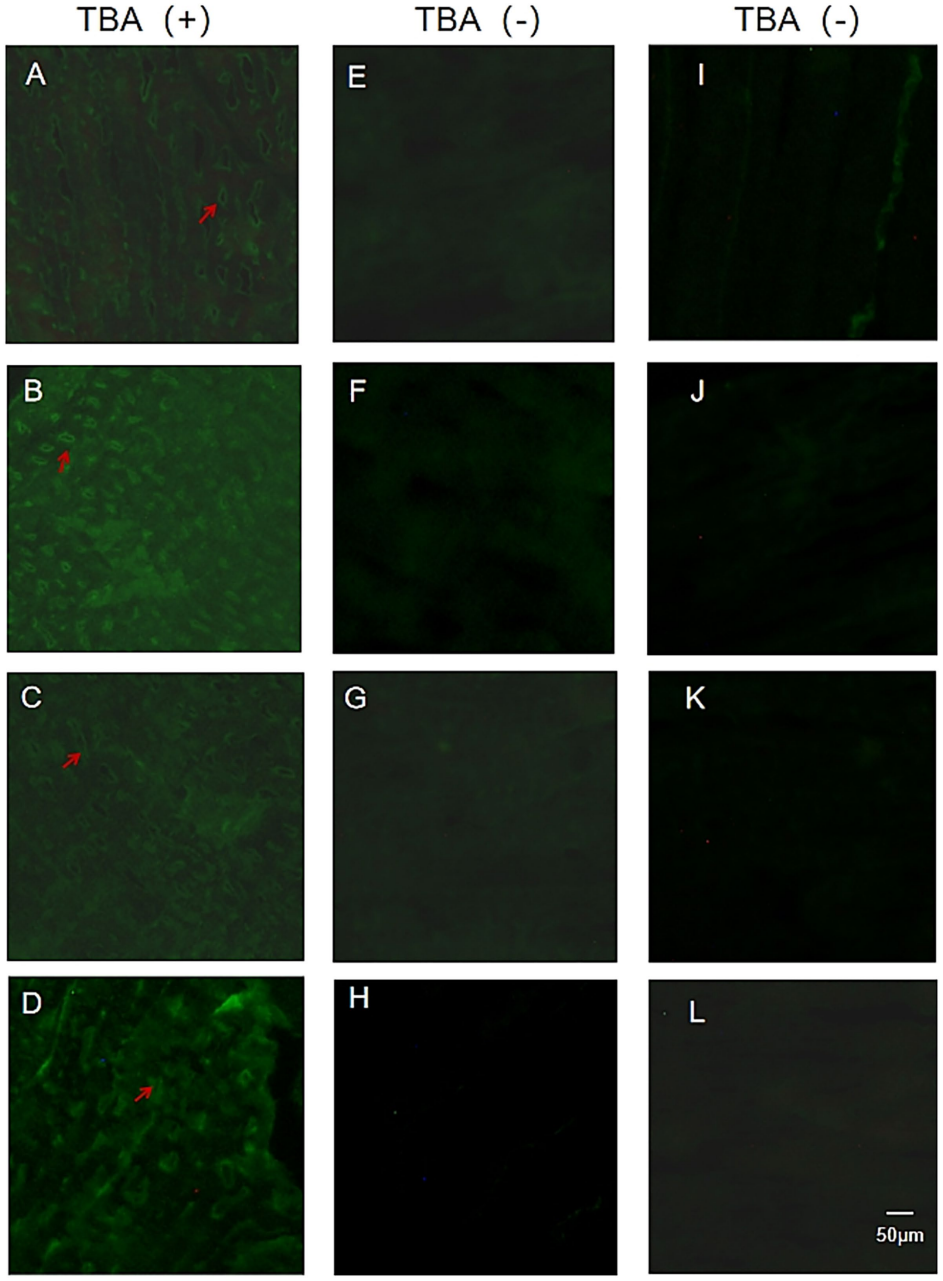
TBA fluorescence maps of SNMG patients and healthy controls. **(A–D)** Representative images of TBA showing characteristic fluorescence signals in four SNMG patients. **(E–H)** Representative images of TBA showing absent characteristic fluorescence signals in four SNMG patients. **(I–L)** Representative images of TBA showing absent characteristic fluorescence signals in four healthy controls.

Tissue immunofluorescence detection using frozen sections of rat gastrocnemius muscle exhibited that 4 out of 20 SNMG patients presented characteristic circle-like fluorescence signals, as shown in Figure 1. In the serum of these four SNMG patients, RyR-Ab and titin-Ab were negative, indicating that unknown autoantibodies were targeting muscle proteins.

### 3.2 Identification of ALDOA as an MG-associated autoantigen

**Figure 2:**
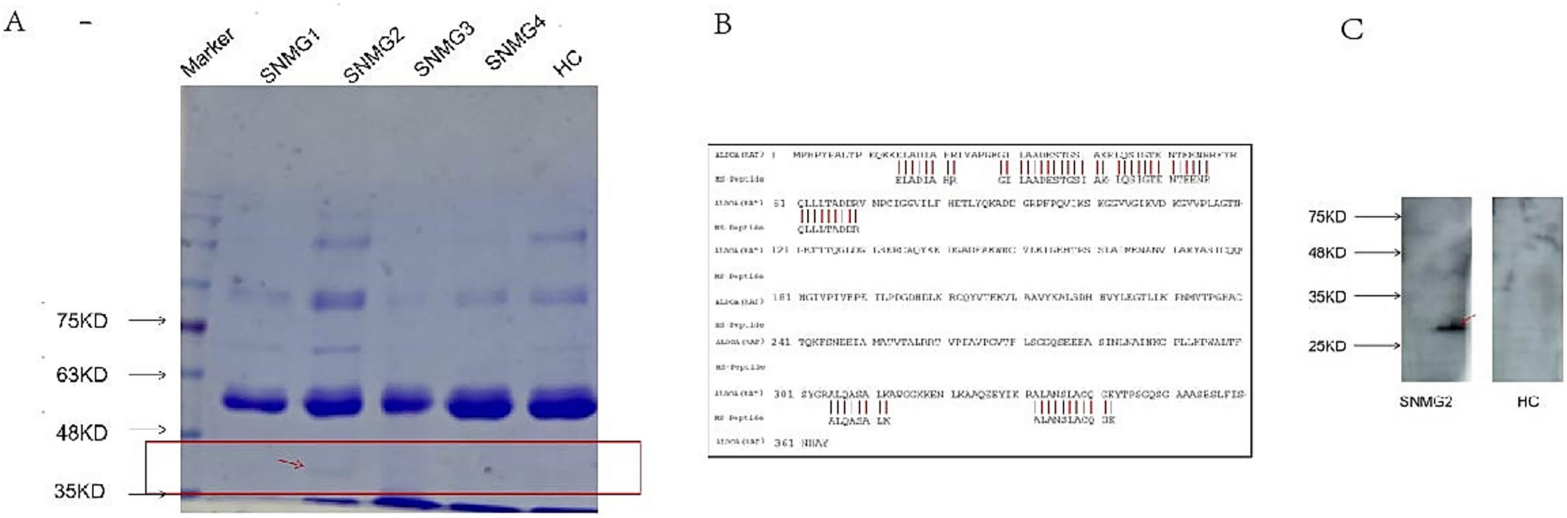
SNMG group-specific reaction bands. **(A)** SDS-PAGE analysis showing a specific band at approximately 40 kD in the immunoprecipitation products of rat gastrocnemius muscle protein incubated with serum from an SNMG patient (lane 1: protein marker; lane 2: immunoprecipitated sample). **(B)** Mass spectrometry identification of the excised protein band (indicated by the red arrow in A) as Rattus norvegicus Aldolase A (ALDOA). **(C)** Western blot validation using commercial human ALDOA protein, confirming the presence of anti-ALDOA antibodies in the serum of the SNMG patient.

The immunoprecipitation products of rat gastrocnemius muscle protein and serum from an SNMG patient showed specific bands at 40 kilodaltons (kD) on SDS-PAGE (Figure 2A). The protein was identified as *Rattus norvegicus* (Rat) ALDOA by mass spectrometry after excising the strip (Figure 2B), and the presence of ALDOA-Ab was verified in the serum of the SNMG patient using the commercialized human ALDOA protein (Figure 2C).

### 3.3 Diagnostic specificity of ALDOA-ab

**Figure 3:**
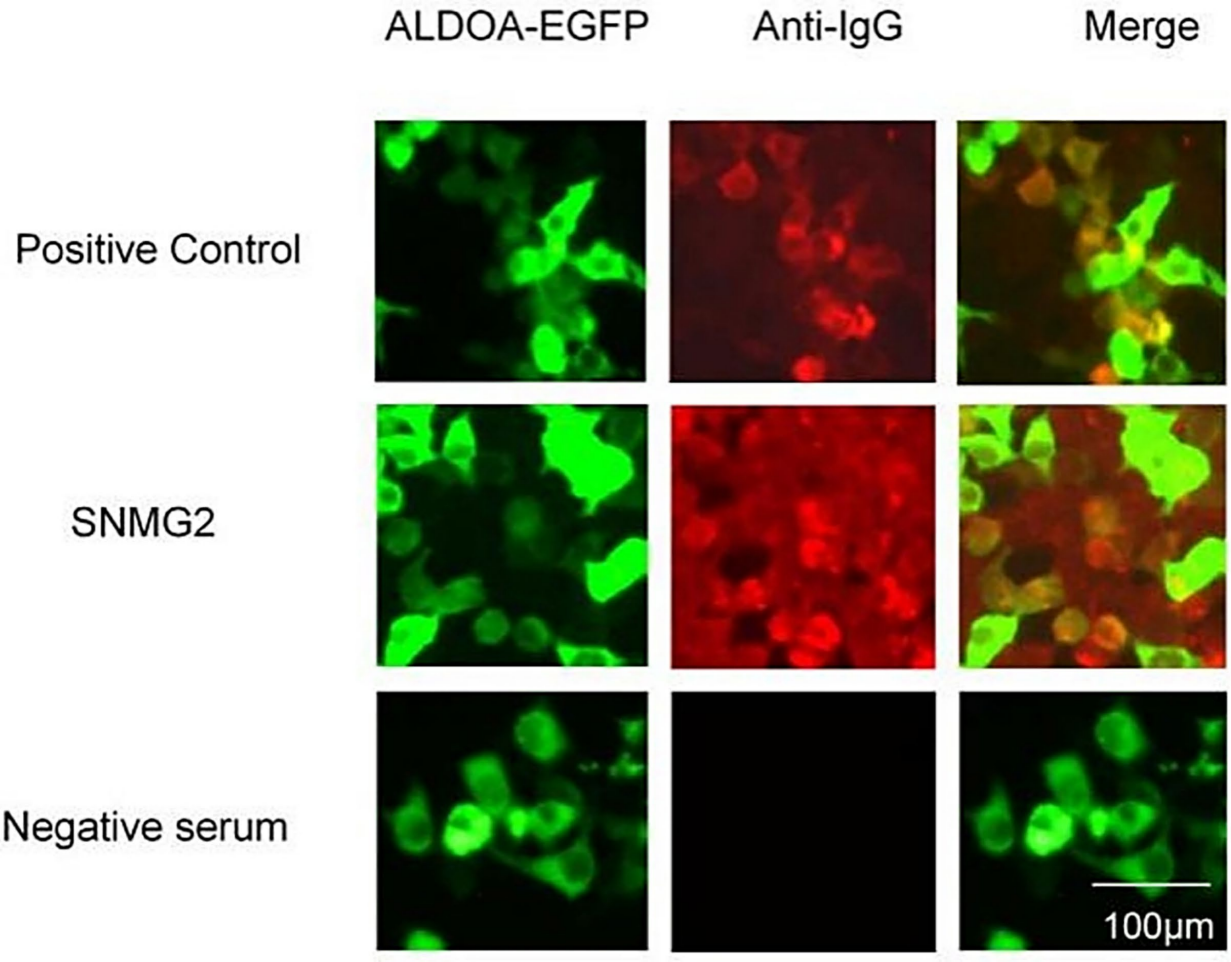
CBA detection and Western blotting validation of ALDOA-Ab-positive patients during the screening phase. **(A)** CBA detection of ALDOA-Ab in the serum of an SNMG 2 patient; **(B)** Western blotting validation of ALDOA-Ab in the serum of an SNMG 2 patient, with the positive reactive band indicated by the red arrow. (NC: negative control transfected with empty plasmid; EGFP: eukaryotic expression of pcDNA3.1-EGFP-C vector protein; ALDOA-EGFP: eukaryotic expression of pcDNA3.1-ALDOA-EGFP fusion protein).

A CBA method was established for detecting ALDOA-Ab. The serum of SNMG patients was tested, and the SNMG2 patient was identified as positive for ALDOA-Ab, as shown in Figure 3A, consistent with the serum proteomics results. Western blotting analysis of the patient’s serum using eukaryotically expressed ALDOA protein (Figure 3B) revealed a positive band (indicated by the red arrow), further confirming that the patient was serum ALDOA-Ab positive. Immunoprecipitation-based serum proteomics was utilized to screen for the presence of ALDOA-Ab in the sera of patients with SNMG, and a CBA method was established for the detection of ALDOA-Ab in HC and IIM sera. ALDOA-Ab was not detected in the sera of 80 HC and 20 IIM controls, indicating that it is specific for the diagnosis of SNMG.

### 3.4 Serum ALDOA-ab in patients with SPMG

**Figure 4:**
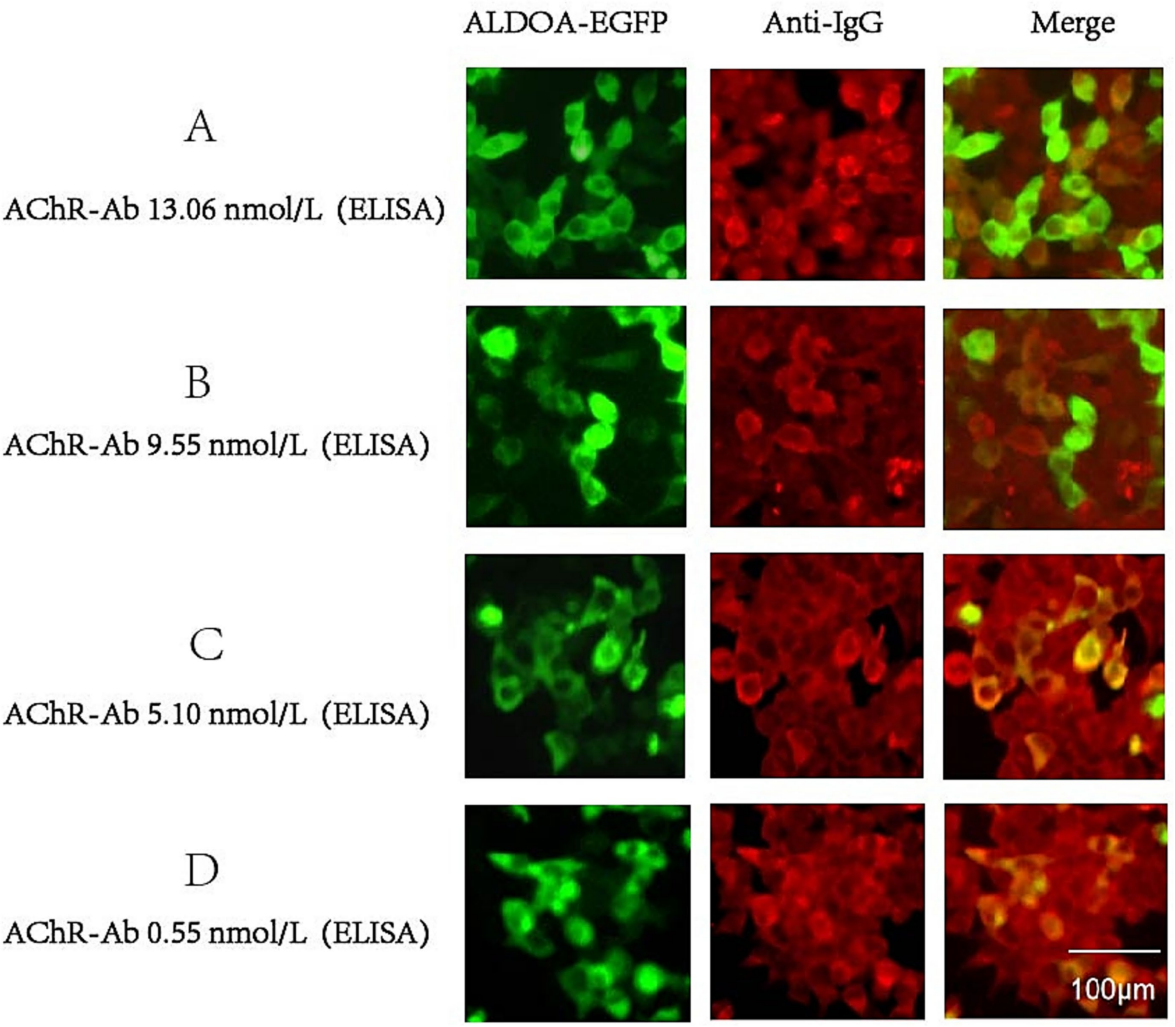
CBA results of ALDOA-Ab-positive SPMG patients. **(A-D)** Representative immunofluorescence images showing ALDOA-Ab detection in sera from AChR-Ab-positive MG patients with varying AChR-Ab titers: **(A)** 13.06 nmol/L, **(B)** 9.55 nmol/L, **(C)** 5.10 nmol/L, and **(D)** 0.55 nmol/L. Scale bar: 100 μm.

We screened the serum of 656 SPMG patients for ALDOA-Ab using the CBA method and found that ALDOA-Ab can also be detected in the serum of SPMG patients. Representative CBA results confirming the presence of ALDOA-Ab in sera from AChR-MG patients are shown in Figure 4.

**Figure 5:**
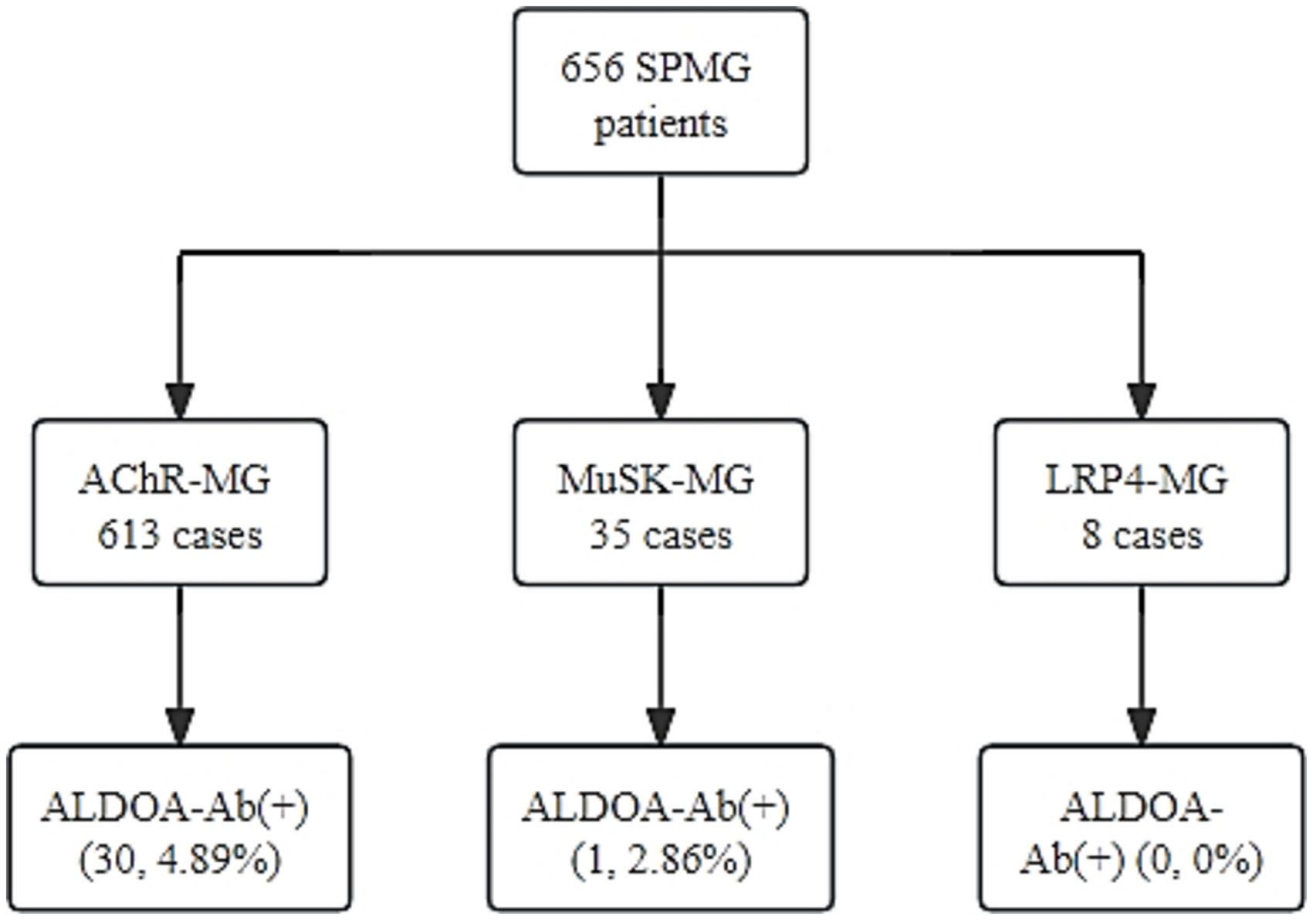
The level of ALDOA-Ab in the serum of SPMG patients.

According to the serological grouping of MG patients, those with single-positive AChR-Ab, MuSK-Ab, and LRP4-Ab were defined as AChR-MG, MuSK-MG, and LRP4-MG, respectively, and ALDOA-Ab positivity was observed in 30 out of 613 AChR-MG patients, with a positivity rate of 4.89%. In 35 MuSK-MG patients, one was positive for ALDOA-Ab, with a positivity rate of 2.86%. ALDOA-Ab was not detected in the LRP4-MG subgroup, and the highest rate of ALDOA-Ab positivity was found in the AChR-MG subgroup, as shown in detail in Figure 5. These findings indicate that ALDOA-Ab not only existed alone in the serum of SNMG patients but also coexisted with other antibodies in the serum of SPMG patients.

### 3.5 ALDOA-ab subtype identification

**Figure 6:**
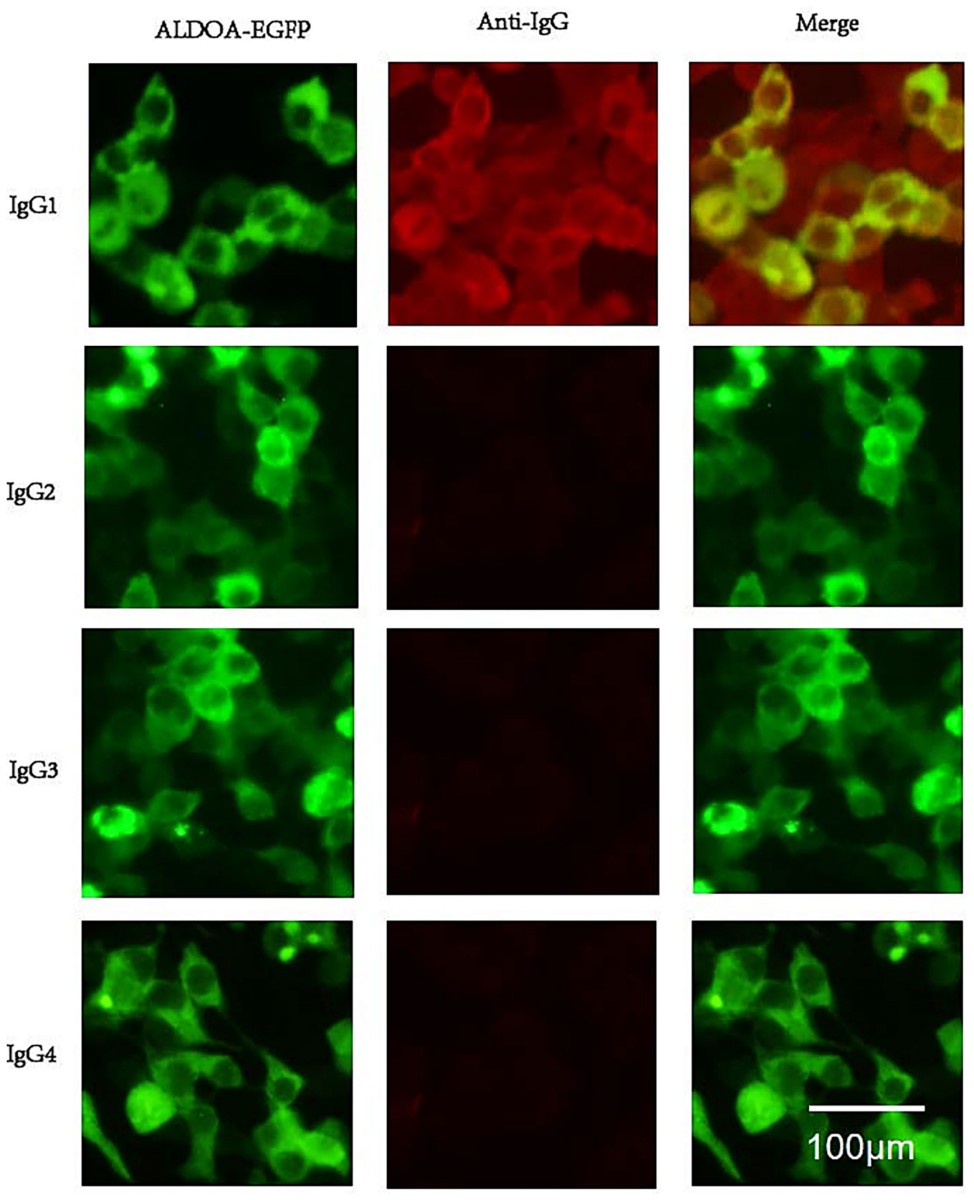
ALDOA Ab subtype phenotype. Serum from an anti-ALDOA-positive MG patient was analyzed by cell-based assay using HEK293 cells expressing ALDOA-EGFP (green). IgG subclasses were detected with subclass-specific secondary antibodies and Alexa Fluor 568-conjugated anti-mouse IgG (red). Top: ALDOA-EGFP (green), anti-human IgG (red), and merged image showing co-localization (yellow). Bottom: IgG subclass staining revealed specific reactivity only for IgG1 (yellow); no signal was detected for IgG2, IgG3, or IgG4. Scale bar: 100 μm. Anti-ALDOA antibodies were predominantly IgG1.

Using the CBA method, the serum from the patient with ALDOA-Ab single-positive MG was found to contain antibodies of IgG1 subtype, and the results of the CBA test have been depicted in Figure 6.

## 4 Discussion

**Figure 7:**
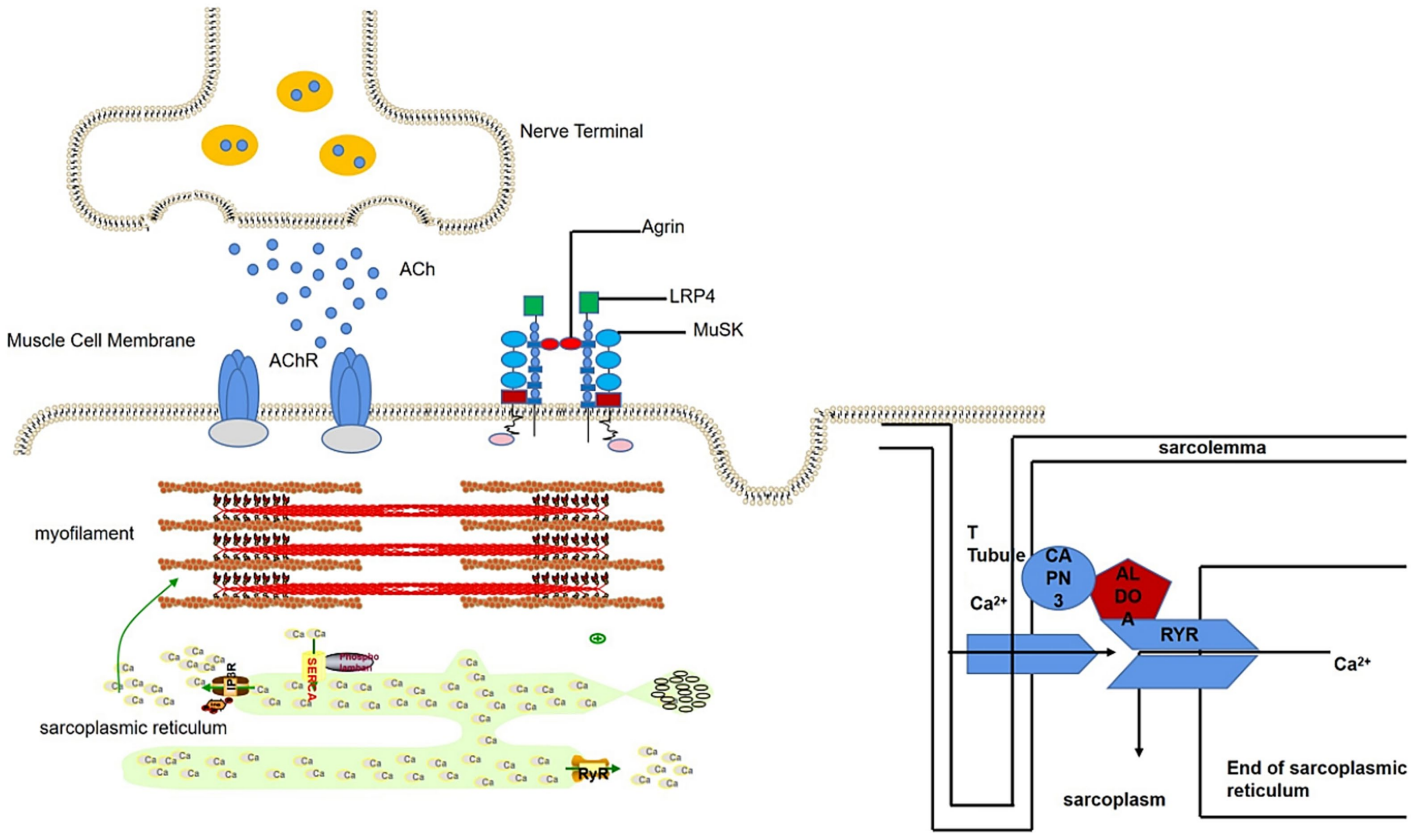
Schematic diagram of the location of ALDOA protein at the neuromuscular junction.

We detected ALDOA-Ab in the serum of SNMG patients through Western blotting but not in healthy controls or IIM disease controls. ALDOA is a glycolytic enzyme widely expressed in tissues, particularly in developing and adult muscle, where it supports cellular functions and muscle-related processes (15). As illustrated in Figure 7, ALDOA localizes to myocytes at the neuromuscular junction (NMJ) and is associated with muscle triads (16): structures formed by T-tubules and the sarcoplasmic reticulum (SR), which enable excitation-contraction coupling via Ca^2+^ release (17, 18). ALDOA binds to the trichoblasts to produce adenosine triphosphate (ATP), which is required for Ca^2+^ uptake into the SR after excitation (19, 20), replenishing depleted sarcoplasmic reticulum calcium stores in preparation for the next muscle contraction. In addition, ALDOA interacts directly with the RyR, regulating RyR activity and influencing Ca^2+^ release (21). Mutations in the ALDOA gene are known to cause hereditary myopathy and hemolytic anemia, characterized by muscle weakness and exercise intolerance, symptoms that parallel the fatigability seen in MG patients with ALDOA-Ab (22). ALDOA protein affects muscle function by influencing Ca^2+^ release and recycling. It is therefore plausible that the presence of ALDOA-Ab could lead to a functional deficiency of ALDOA protein, potentially impairing Ca^2+^ handling at the NMJ and contributing to muscle weakness. However, whether ALDOA-Ab is directly pathogenic or merely a secondary phenomenon remains to be determined (23).

Analysis of the distribution of ALDOA-Ab in various serological subgroups of SPMG patients revealed that ALDOA-Ab could coexist with AChR-Ab and MuSK-Ab. We did not find ALDOA-Ab in LRP4-Ab-positive patients, probably due to the small sample size (only eight patients). Three main reasons for the coexistence of two related antibodies in the serum of MG patients have been identified. The first possibility may be related to epitope spreading, where inflammation or tissue damage following thymectomy in MG patients exposes cryptic antigenic epitopes, inducing an immune response and leading to the production of additional autoantibodies. The second possibility is the loss of immune tolerance due to changes in the molecular structure of the self, inducing an immune response that produces autoantibodies. The third possibility is that when antibodies cause tissue damage, they expose antigenic epitopes of related proteins, generating an immune response and antibody coexistence (24). The detection of multiple antibodies can improve the diagnosis rate of MG (3, 25, 26).

According to data from the Human Protein Atlas,^1^ ALDOA mRNA is abundantly expressed in skeletal muscle (27). Prelaboratory analysis of protein expression changes in MG intercostal muscles using isobaric tandem mass tag (TMT)-labeled proteomics revealed that ALDOA protein expression was significantly lower in MG patients (*p*-value < 0.01). We hypothesize that the observed reduction in ALDOA protein expression in MG muscle tissue could either promote disease progression by causing ALDOA insufficiency or, alternatively, be a consequence of protein degradation secondary to NMJ injury. Both scenarios suggest a potential, though not yet causative, link between ALDOA and MG. Consequently, the primary question remains whether ALDOA-Ab is a main pathogenic driver of MG or a secondary biomarker resulting from epitope spreading following NMJ damage. If ALDOA-Ab is pathogenic, it could cause ALDOA protein insufficiency and exacerbate the disease. If it is non-pathogenic, its presence might simply be a consequence of tissue injury exposing intracellular ALDOA antigen. Elucidating the pathogenic role of ALDOA-Ab is therefore essential to determine its clinical significance.

This study has several limitations. First, the pathogenicity of ALDOA-Ab remains unproven, and its status as an intracellular target suggests that it may be a biomarker rather than a direct cause of pathology. Second, the sample size, particularly for the SNMG subgroup and disease controls (IIM), was relatively small, which may affect the generalizability of our findings. Third, while we used a highly specific CBA for MuSK, LRP4, and ALDOA-Ab detection, an ELISA was used for AChR-Ab detection during initial screening. Although ELISA is a standard method for AChR-Ab detection, its sensitivity is lower than that of CBA, and some AChR-Ab-positive MG patients may have been missed. Future studies with larger, multicenter cohorts and uniform high-sensitivity antibody testing methodologies are warranted to confirm our observations.

To further elucidate the role of ALDOA-Ab in MG, future research should focus on several key areas. First, passive transfer experiments, wherein purified ALDOA-Ab from patients is injected into animal models, are essential to definitively assess its pathogenicity. Second, larger-scale multicenter studies are required to validate the diagnostic and prognostic value of ALDOA-Ab and to firmly establish its associated clinical phenotype. Third, investigating the mechanisms that trigger the anti-ALDOA immune response, such as viral infections or specific genetic backgrounds, could provide insights into the disease onset. Finally, exploring the potential of ALDOA-Ab as a biomarker for predicting progression from ocular to generalized MG could have significant clinical utility.

In summary, we identified ALDOA-Ab in the sera of MG patients, particularly within the SNMG subgroup. While its pathogenicity requires further investigation, ALDOA-Ab shows promise as a novel diagnostic biomarker. Hence, future studies are needed to validate these findings and explore its pathophysiological role.

## Data Availability

The raw data supporting the conclusions of this article will be made available by the authors, without undue reservation.

## Footnotes

^1^ https://www.proteinatlas.org/
